# Pedigree association: assigning individual weights to pedigree members for genetic association analysis

**DOI:** 10.1186/1753-6561-3-s7-s121

**Published:** 2009-12-15

**Authors:** Stacey Knight, Ryan P Abo, Jathine Wong, Alun Thomas, Nicola J Camp

**Affiliations:** 1 Biomedical Informatics Department, University of Utah, 26 South 2000 East Room 5775 HSEB, Salt Lake City, Utah 84112, USA

## Abstract

**Objective:**

Methods exist to appropriately perform association analyses in pedigrees. However, for genome-wide association analysis, these methods are computationally impractical. It is therefore important to determine alternate methods that can be efficiently used genome-wide. Here, we introduce a new algorithm that considers all relationships simultaneously in arbitrary-structured pedigrees and assigns weights to pedigree members that can be used in subsequent analyses to address relatedness. We compare this new method with an existing weighting algorithm, a naïve analysis (relatedness is ignored), and an empirical method that appropriately accounts for all relationships (the gold standard).

**Methods:**

Framingham Heart Study Genetic Analysis Workshop 16 Problem 2 data were used with a dichotomous phenotype based on high-density lipoprotein cholesterol level (1,611 cases and 4,043 controls). New and existing algorithms for calculating weights were used. Cochran-Armitage trend tests were performed for 17,333 single-nucleotide polymorphisms on chromosome 8 using both weighting systems and the naïve approach; a subset of 500 single-nucleotide polymorphisms were tested empirically. Correlations of *p*-values from each method were determined.

**Results:**

Results from the two weighting methods were strongly correlated (*r *= 0.96). Our new weighting method performed better than the existing weighting method (*r *= 0.89 vs. *r *= 0.83), which is due to a more moderate down-weighting. The naive analysis obtained the best correlation with the empirical gold standard results (*r *= 0.99).

**Conclusion:**

Our results suggest that weighting methods do not accurately represent tests that account for familial relationships in genetic association analyses and are inferior to the naïve method as an efficient initial genome-wide screening tool.

## Background

Many researchers have already ascertained and collected DNA on large pedigrees for linkage studies. The ability to use these familial cases in association studies is appealing not only because of the decrease in ascertainment cost, but also because these cases are more likely to be due to a genetic cause and therefore can be more informative for genetic research [1]. However, the use of these cases results in correlated observations and treating these cases and controls as independent will increase the false-positive rate [2].

There are a few methods developed for association testing in pedigrees of arbitrary size. One approach is to assume independence for the calculation of the point estimate of the random variable of interest and to adjust the variance, hence altering the statistic or confidence intervals [3]. However, currently this method has only been developed for a limited number of test statistics. Another approach is to use generalized estimating equations (GEE). However, GEE does not take into account the full pedigree structure in the development of the correlation matrix and has been shown to fail due to singularity in the correlation matrix and sparse data [4]. Empirical methods, which can applied to a wide variety of statistics, have been developed for association testing in pedigrees [5]. While empirical methods address several of the issues associated with the GEE and variance-correction methods, the number of simulations required in these methods to reach genome-wide significance is too time intensive for a complete genome-wide association study.

An approach for screening the whole genome using arbitrarily structured pedigrees that is efficient and provides accurate and reliable estimates is needed. Assigning weights, which are designed to account for familial correlation, to pedigree members appears to be a promising approach. If appropriate weights can be established, then analyses of related individuals could proceed using those standard statistical techniques that allow incorporation of weights. Because many statistical methods allow for weights (e.g., chi-square test and regression analysis), the use of weights has the flexibility of both qualitative and quantitative traits and allows for the examining of different genetic patterns (e.g., dominance and maternal effects). Previously, an algorithm was proposed that used pairwise relationships to determine weights [6]. The purpose of this paper is to introduce a new weighting algorithm that considers all relationships simultaneously and to examine the use of both weighting algorithms as methods for association testing in pedigrees of arbitrary size. We also compare findings with a naïve analysis in which cases and controls in pedigrees were treated as independent (that is, familial relationships were simply ignored), and an empirical method that appropriately accounts for all relationships and is used as the gold standard.

## Methods

### Study population and phenotype

We used the real Framingham Heart Study data (Genetic Analysis Workshop 16 Problem 2). This dataset had 6,752 subjects with both phenotype and genotype data. Most (96.6%) of these individuals reside in pedigrees with three generations at most. We defined a dichotomous phenotype based on high-density lipoprotein cholesterol level (HDL) at first examination. Individuals were considered to be low (cases) or high (controls) for HDL based on a threshold of <50 mg/dL for women and <40 mg/dL for men [7]. Those on cholesterol treatment at the time of the exam were excluded from the analysis. We further excluded individuals with more than 2% missing genotype data. This resulted in a sample size of 5,654 individuals with phenotype and genotype information (1,611 cases and 4,043 controls) and most (92.7%) resided in a pedigree, with an average relatedness closer than avunculars (average coefficient of kinship = 0.165).

### Genotyping

We used chromosome 8 data from the Affymetrix GeneChip^® ^Human Mapping 500 k Array Set and excluded any single-nucleotide polymorphism (SNP) that failed Hardy-Weinberg equilibrium (*p *< 0.001), that had greater than 2% missing data, and had a minor allele frequency of less than 1%. We further removed SNPs that had sparse data and thus were not valid for use in chi-square trend testing. This resulted in a total 17,333 SNPs for analysis.

### Weighting algorithms

The previously published weighting algorithm of Browning et al. is based on pairwise measures of relatedness [6]. That is, each relative pair is considered separately, even if multiple, and non-independent, pairs exist in the same pedigree. This method also excludes all controls from pedigrees that contain cases. The inverse of the prior kinship coefficients matrix is used. Each pedigree member considered in the analysis is assigned half the column sum of that matrix as their weight. The Browning method has been implemented in the software CCREL, which was used to extract weights for pedigree members.

**Figure 1:**
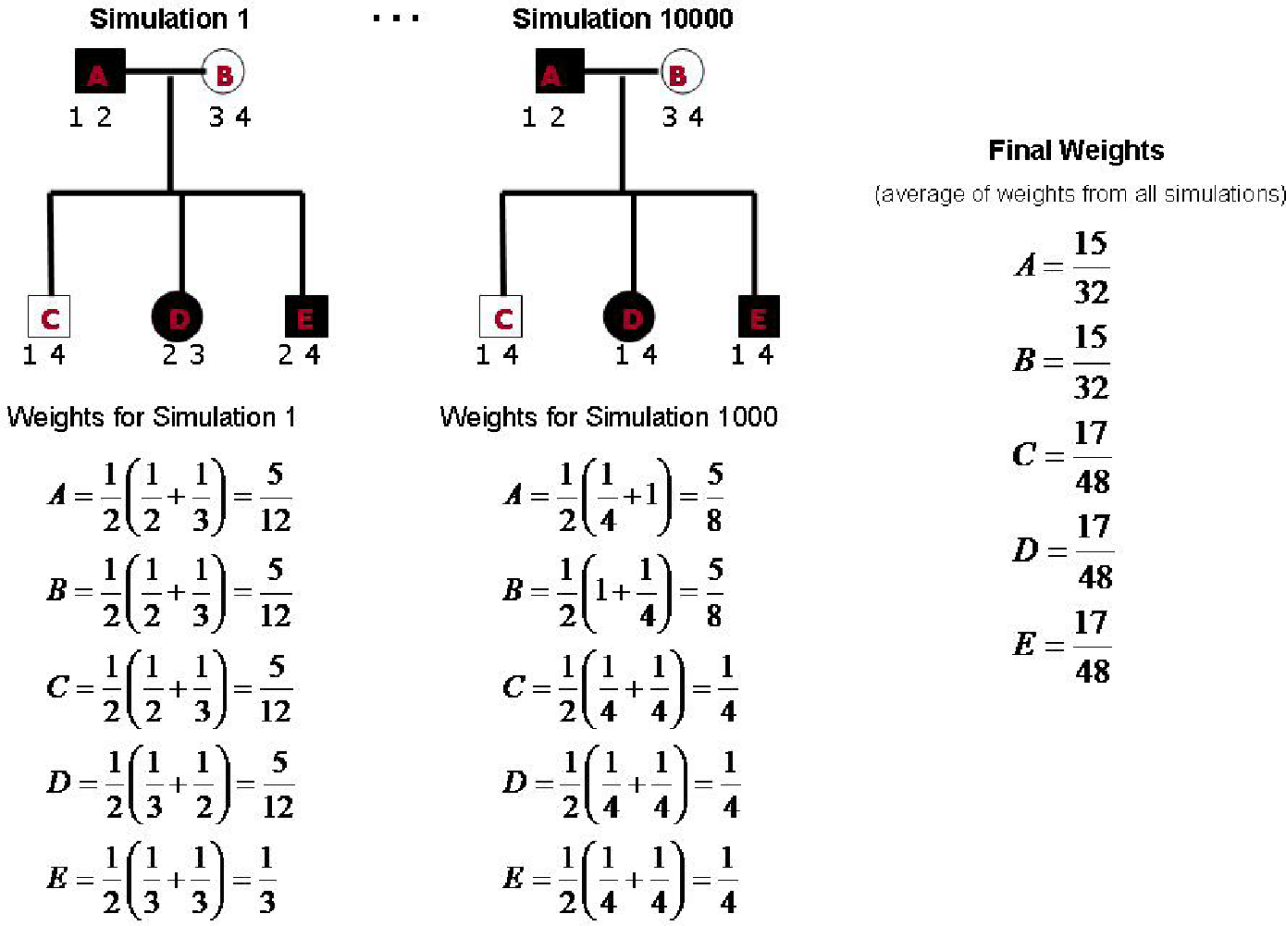
**Weighting simulations for a small nuclear family**.

We developed a new method to assign weights to individuals that simultaneously accounts for all relationships. We use simulation to determine the weights as follows: 1) unique alleles are assigned to all founders (For example, the first founder is given alleles 1 and 2, the second founder 3 and 4, the third founder 5 and 6 and so forth.), 2) a gene-drop is performed (simulated mendelian inheritance), 3) pedigree members weights were assigned as the average of the reciprocals of the number of times each of their alleles were observed in pedigree members to be used in the subsequent analysis, 4) step 3 was repeated 10,000 times and weights were averaged across simulations. Figure 1 contains an illustration of this process. We used simulations because the determining of all possible proportions of allele sharing for all individuals in a pedigree simultaneously is an intractable problem in large arbitrary structured pedigrees.

Two data sets were tested: a "full" data set for which weights were determined for all pedigree members that were to be used in a subsequent association analysis; and a "no pedigree control" set, which excluded controls that were in pedigrees with cases, and weights were re-calculated omitting these controls. The method of Browning et al. was only designed for the latter scheme and therefore is considered only in the "no pedigree control" comparisons.

### Statistical analysis

Cochran-Armitage trend tests were performed on both the "full" and "no pedigree control" data sets using a naïve test (ignoring relationships) and with our new weights and Browning's weights (no pedigree control data only) for all markers. SAS was used to perform the weighted and naïve trend tests. Five hundred SNPs were randomly selected and empirical, gold standard trend tests were performed using the software package PedGenie [5]. The empirical *p*-values were based on 100,000 simulations for the null distribution. Correlations of the *p*-values resulting from each method were calculated. For ease in presentation, the -log_10 _(*p*-values) are plotted for the figures.

## Results

### Chromosome 8 results

There were 68 negative weights assigned using the method of Browning et al. (49 of which were essentially zero, ≤3 × 10^-15^). Because standard weighting techniques (including SAS) will not allow negative weights, these were set to have zero weight. Our new weighting method always results in positives scores between 0 and 1. The effective total sample size (sum of weights) for our new method was 1,961 compared with 1,802 for the method of Browning et al. The average difference between the weights for the two algorithms was 0.012 (standard deviation of 0.071).

The distributions of *p*-values for the Cochran-Armitage trend test for the "full" and "no pedigree controls" data sets and by the different methods are shown in Table 1. For all methods, the use of the full data set resulted in fewer significant *p*-values (*p *< 0.05) compared with the "no pedigree control" set, thus raising the question regarding study design for selection of controls, although for the empirical method the difference was small (147 and 144). For both data sets, the naïve method resulted in the largest number of significant *p*-values. For the "no pedigree control" data set, both weighting methods had a similar distribution of *p*-values.

**Table 1:** Summary of the distribution of *p*_trend _values

	Full			No pedigree controls			
		
***p***_**trend**_	New weights	Naïve	Empirical	Browning weights	New weights	Naïve	Empirical
*N *= 17,333							
<1 × 10^-4^	11	22	NA	15	15	108	NA
1 × 10^-4 ^≤ *p*<1 × 10^-3^	4	48	NA	28	44	186	NA
1 × 10^-3 ^≤ *p *< 0.01	51	333	NA	186	240	723	NA
0.01 ≤ *p *< 0.05	333	985	NA	695	816	1,615	NA
Total < 0.05	399	1,388	NA	924	1,115	2,632	NA
≥0.05	16,933	15,945	NA	16,409	16,218	14,712	NA
*N *= 500							
<1 × 10^-4^	0	2	1	1	1	21	1
1 × 10^-4 ^≤ *p*<1 × 10^-3^	2	9	2	13	19	33	6
1 × ^-3 ^≤ *p *< 0.01	14	56	33	32	49	101	36
0.01 ≤ *p *< 0.05	68	139	108	134	132	162	104
Total < 0.05	84	206	144	180	201	317	147
≥0.05	416	294	356	320	299	183	353

**Figure 2:**
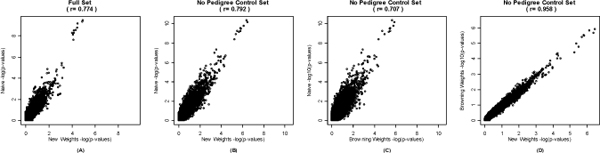
**Chromosome 8 -log_10 _(*p*_trends_) values for the different methods**.

The *p*-values from the naïve and weighting methods for both data sets were highly correlated (all *r *> 0.7), although substantial variance is observed (Figure 2). The naïve method most often resulted in smaller *p*-values: between 71.1-71.8% of the *p*-values for the SNPs compared with any weighting method/data set combination. A stronger correlation with less spread was observed between the two weighting methods (*r *= 0.96; Figure 2D). The correlation of the *p*-values did not change when stratified by minor allele frequency.

### Empirical results

**Figure 3:**
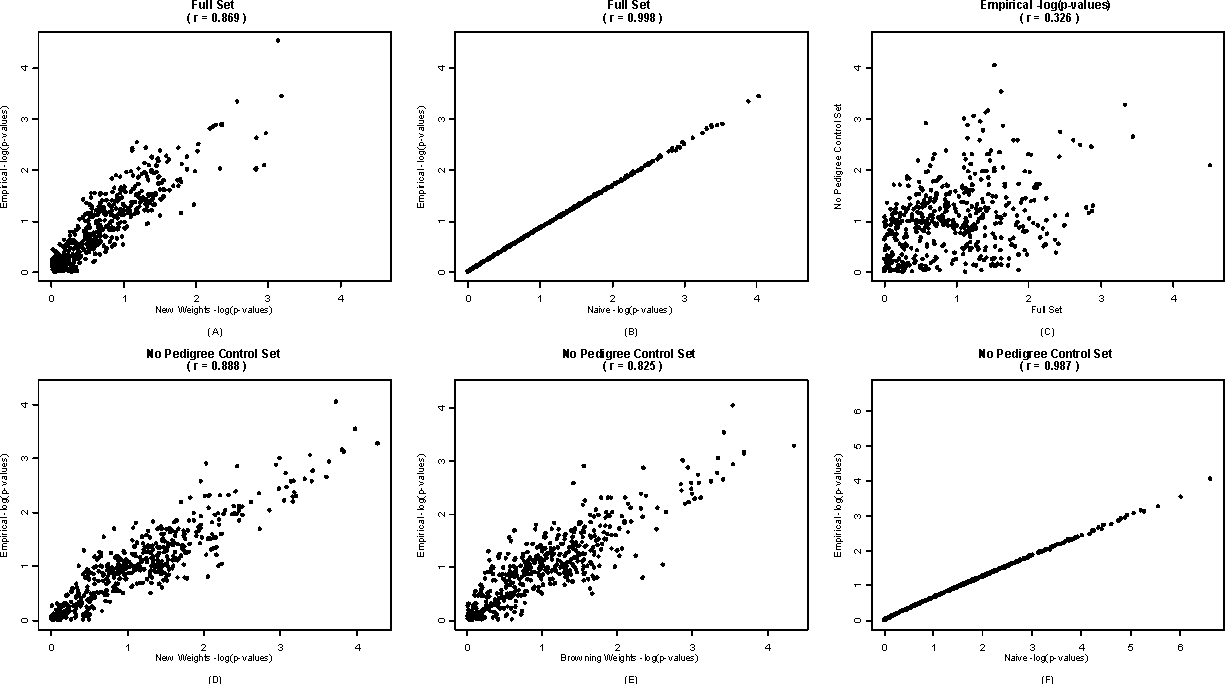
**The -log_10 _(*p*_trends_) values empirical comparisons**. Reported correlations are based on original *p*_trends _and not the -log-transformed values.

Figure 3 shows the correlations between the empirical values and all other methods for 500 SNPs. The naïve *p*-values have the greatest correlation with the gold standard empirical *p*-values for both data sets (*r *= 0.998 and *r *= 0.987; Figure 3B and 3F). In addition, a strong unidirectional relationship was observed. For both data sets, the naïve *p*-values were smaller than the empirical *p*-values.

While our new weighting method and the method of Browning et al. produced similar *p*-values (*r *= 0.96; Figure 2D), our new method appeared to be slightly more correlated with the empirical *p*-values (*r *= 0.888 vs. *r *= 0.825, Figure 3D and 3E). However, neither weighting method obtained a correlation with the gold standard as high as the naive method and neither exhibited a unidirectional relationship with the empirical method as consistent as the naïve method. For the full data, comparing *p*-values from our new weighting method to the empirical results, 28.4% of the *p*-values from our weighting method were smaller than the empirical method *p*-values, the remaining 71.6% were larger. For the "no pedigree control" data set, 62.8% of the *p*-values from our weighting method were smaller and 37.2% were larger than the equivalent empirical *p*-values, and the Browning method had 54.2% *p*-values that were smaller and 45.8% that were larger.

The correlation between the empirical results from the full data set and the "no pedigree control" data set was low (*r *= 0.326, Figure 3C), with no strong unidirectional relationship: 57% of the time the "no pedigree control" empirical *p*-value was less than the full set empirical *p*-value.

## Discussion

In this study we found that our new weighting method performed similarly to the weighting method of Browning et al. (*r *= 0.96), but had the advantages of always generating positive weights; producing higher effective sample size (1,961 vs. 1,802), which will increase power; and resulting in a stronger correlation with the empirical *p*-values. The increase in effective sample size observed is most likely because pairwise methods can overly down-weight multiple individuals belonging to the same pedigree. Results from both weighting methods were in high correlation with results from both the naïve and empirical approaches. However, the naïve approach had almost perfect correlation with empirical results, and while always anti-conservative, the high correlation and consistency in directional difference arguably make it a superior alternative to either weighting method for a first-pass screening method.

There are limitations of this study that may have lead to the naïve approach performing the best in these analyses. The average pairwise kinship coefficient for the pedigree members was 0.165, indicating that on average the relationships were closer than avunculars; however, this may not be sufficient relatedness to illustrate the strength of methods that attempt to adjust for related individuals. Second, the weights assigned are based on expected sharing and hence this will differ from observed sharing on a SNP-by-SNP basis. Thus, weights could both under- and over-estimate specific SNP-sharing producing more varied results. Another limitation is that we have examined only a qualitative trait. Because quantitative trait analysis is often more powerful than a qualitative analysis, it is possible that correcting for familial relationships would have a greater affect. Also, we have only examined familial correlation to adjust for inflated *p*-values and have not examined other sources of inflation such as population stratification. Finally, while it has been suggested that using individual weights can effectively adjust for familial correlations in analyses, this may be an over-simplification of the problem.

In a previous study it was found that the method of Browning et al. was valid and had power similar to variance correction methods [6]. However, the lower correlation of the weighting tests with the empirical approach and the fact that weighting methods can appear both conservative and anti-conservative compared with the empirical (see Table 1) indicate that weighting methods are not providing equivalent tests to those available in previously validated methods for pedigree association testing [3,5]. Hence, any equivalence of power that was previously noted [6] may not be due to an equivalence of the tests, and it could be that the "no pedigree control" design adopted in our application of the method of Browning et al. is superior. We found that the empirical *p*-values from the "full" and "no pedigree control" data sets were not highly correlated and that more significant *p*-values were generated in the "no pedigree control" design. Further work is needed to clarify the role that weighting methods can play and to elucidate optimal study designs.

## Conclusion

Our results suggest that association tests using weights do not accurately represent tests that extend standard genetic association methods to familial relationships in pedigree data. Furthermore, they are inferior to the naïve method for a screening tool. The high correlation and unidirectional relationship between the empirical and naïve methods suggests that the naïve method is the superior way to efficiently perform a first-pass screen for association analyses in pedigree data. Due to the anticonservative nature of the naïve method, however, significant thresholds may need to be lowered for the initial screen, and the results that surpass thresholds from the first-pass must be followed by a second-step analysis that can accurately determine significance by accounting for the familial structure. This two-step approach is efficient and will lead to results that are equivalent to a full primary screen using tests that account for familial relationships in genetic association analyses.

## List of abbreviations used

GEE: Generalized estimating equations; HDL: High-density lipoprotein; SNP: Single-nucleotide polymorphism.

## Competing interests

The authors declare that they have no competing interests.

## Authors' contributions

All authors contributed to the design, concept, writing, and editing of the paper.
